# Analysis of saccharification in *Brachypodium distachyon *stems under mild conditions of hydrolysis

**DOI:** 10.1186/1754-6834-1-15

**Published:** 2008-10-22

**Authors:** Leonardo D Gomez, Jennifer K Bristow, Emily R Statham, Simon J McQueen-Mason

**Affiliations:** 1 CNAP, Department of Biology, University of York, Heslington, York YO10 5YW, UK

## Abstract

**Background:**

*Brachypodium distachyon *constitutes an excellent model species for grasses. It is a small, easily propagated, temperate grass with a rapid life cycle and a small genome. It is a self-fertile plant that can be transformed with high efficiency using *Agrobacteria *and callus derived from immature embryos. In addition, considerable genetic and genomic resources are becoming available for this species in the form of mapping populations, large expressed sequence tag collections, T-DNA insertion lines and, in the near future, the complete genome sequence. The development of *Brachypodium *as a model species is of particular value in the areas of cell wall and biomass research, where differences between dicots and grasses are greatest. Here we explore the effect of mild conditions of pretreatment and hydrolysis in *Brachypodium *stem segments as a contribution for the establishment of sensitive screening of the saccharification properties in different genetic materials.

**Results:**

The non-cellulosic monosaccharide composition of *Brachypodium *is closely related to grasses of agricultural importance and significantly different from the dicot model *Arabidopsis thaliana*. Diluted acid pretreatment of stem segments produced significant release of sugars and negatively affected the amount of sugars obtained by enzymatic hydrolysis. Monosaccharide and oligosaccharide analysis showed that the hemicellulose fraction is the main target of the enzymatic activity under the modest hydrolytic conditions used in our assays. Scanning electron microscopy analysis of the treated materials showed progressive exposure of fibrils in the stem segments.

**Conclusion:**

Results presented here indicate that under mild conditions cellulose and hemicellulose are hydrolysed to differing extents, with hemicellulose hydrolysis predominating. We anticipate that the sub-optimal conditions for hydrolysis identified here will provide a sensitive assay to detect variations in saccharification among *Brachypodium *plants, providing a useful analytical tool for identifying plants with alterations in this trait.

## Background

Although the contribution of agricultural waste to the generation of transportation fuels has been negligible, in recent years there has been an upsurge of interest in the use of grass straw from agricultural waste as well as dedicated energy crops for the production of biofuels [1]. Since grasses of agricultural importance have complex genomes and growth requirements that make them difficult for use in research at the molecular level, there is a need for model grass species in basic research. This need for grass model species is particularly evident in the area of cell wall research, where dicots and grasses differ substantially in the composition and organisation of the component polymers [2,3]. A growing range of genetic tools and the growth characteristics of *Brachypodium distachyon *make it potentially a good model for grass research [4,5]. A 4× draft of the genome sequence has been released and an 8× assembly will be available [6].

The high costs associated with the three biological steps involved in the conversion of lignocellulose to biofuels (enzyme production, biomass hydrolysis and fermentation of the released sugars) have driven numerous efforts to make the overall biochemical conversion more efficient [7]. These efforts have been directed not only to reduce the costs of enzymes, but also to optimise the configuration of all the steps of the process [8]. Any approach for improving the process of saccharification of lignocellulosic plant biomass requires, or at least will benefit from, a thorough understanding of the structure and biosynthesis of plant cell walls [9]. Using *Brachypodium *as a model for understanding the characteristics of the grass cell wall involved in the process of saccharification will require a detailed characterisation of the saccharification in this material under different conditions [6].

The conversion of lignocellulose into sugars depends on variables present at different levels of organisation in the cell wall, from the diversity of the glycosidic bonds involved in the synthesis of the wall polymers, to the organisation and the relative abundance of each component [10]. Indeed, alfalfa mutants with altered lignin levels present improved digestibility, and saccharification in maize and sorghum is affected by ferulic acid cross-links [11,12].

Industrial procedures for the conversion of lignocellulose into sugars for fermentation use chopped biomass, in which the structure and consequently the architecture of lignocellulose represent another factor affecting conversion. The development of improved lignocellulose saccharification assays, relevant to industrial considerations, should include materials where tissue structure is intact in order to take in account features of the cell wall that will influence digestibility in 'real-life' conditions.

Here we present a characterisation of the saccharification of *Brachypodium *stem segments in order to understand the modifications occurring in lignocellulose under different pretreatment and hydrolysis conditions. We have carried out analyses of the composition of *Brachypodium *stems and the products released, as well as structural changes, occurring in the stem segments under mild conditions of hydrolysis.

## Results

### Monosaccharide composition of *Brachypodium *stems

*Brachypodium *is closely related to agronomically important grasses, making it a potential model species from a phylogenetic point of view [6]. To examine whether *Brachypodium *relatedness to important crop grasses is maintained at the level of cell wall composition, we compared the monosaccharide composition of stem cell wall from several grass species with that of *Arabidopsis*, a well-characterised dicot model.

**Figure 1:**
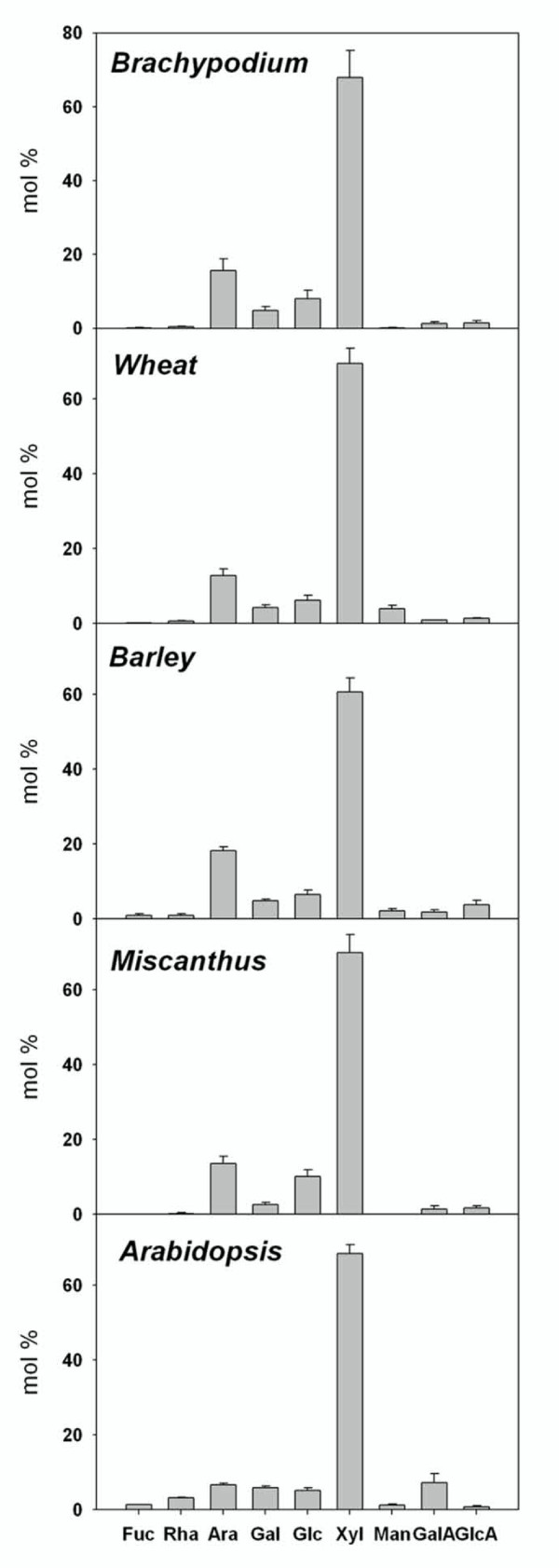
**Non-cellulosic monosaccharide composition of *Brachypodium *stems compared with other species**. Diverging composition is observed between grasses and *Arabidopsis*. Results represent the mean (± SD) of three experiments.

Figure 1 shows that the composition of *Brachypodium *is similar to that of barley, *Miscanthus *and wheat. On the other hand, the monosaccharide composition of the cell wall from the grasses analysed differ significantly from *Arabidopsis*. The percentage of arabinose in grasses is, on average, 2.5 times higher than the amount observed in the dicot species. Conversely, the molar percentage representation of galacturonic acid is around four times and rhamnose around five times higher in *Arabidopsis *than in the grasses. These differences reflect the divergence in polymers constituting the matrix of the cell wall.

Whilst the cell walls of grasses and dicot plants are both largely polysaccharide composites based around a framework of cellulose microfibrils, there are substantial differences between these groups of plants with regard to the composition of the matrix polysaccharides that associate with the cellulose. The primary cell walls of most dicots are relatively rich in pectins, and xyloglucans form the major hemicellulose coating the cellulose microfibrils. In contrast, the primary cell walls of grasses have much less pectin and xyloglucan, and glucuronoarabinoxylan (GAX) and mixed-linkage glucans form the major hemicelluloses [13]. Dicots and grasses also show differences in the composition of secondary cell walls. The hemicellulose of dicot secondary cell walls is typically dominated by simple arabinoxylans with only occasional side chains [14]. In contrast, the GAX of grass secondary cell walls has more complex and numerous side chains, and the arabinosyl side chains may serve as connections to lignin through feruloyl esters [1,14].

The cell walls of dicot species such as *Arabidopsis *are generally much richer in pectic components such as homogalacturonan and rhamnogalacturonan than are grasses, and this is clearly apparent in the higher levels of galacturonic acid released from *Arabidopsis *cell walls. The similarities in non-cellulosic monosaccharide composition between *Brachypodium *and commercially important grasses lend support to the use of *Brachypodium *as a model to explore the conversion of lignocellulose from grasses into ethanol.

### Effect of H_2_SO_4 _concentration and temperature during pretreatment on the hydrolysis of *Brachypodium *stem segments

Many studies have shown the need for pretreatment of the lignocellulosic material in order to produce an efficient hydrolysis [15]. Here we studied different conditions of diluted acid pretreatment in the release of sugars from *Brachypodium *stem segments.

**Figure 2:**
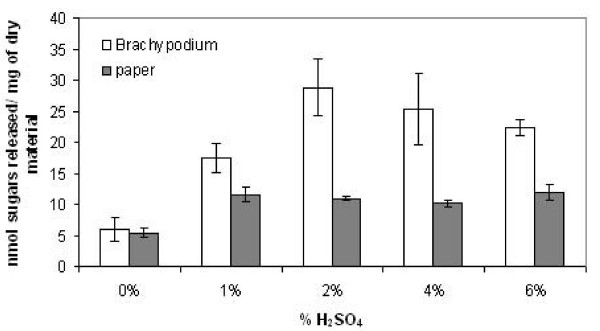
**Sugar release from *Brachypodium *stems and paper using increasing concentrations of H_2_SO_4_**. *Brachypodium *stems and paper discs were pretreated at 90°C in the presence of acid concentration shown in the graph, and subsequently subjected to enzymatic hydrolysis. Results represent the mean (± SD) of three experiments.

Increasing concentrations of H_2_SO_4 _up to 2% at 90°C increased the release of sugars in *Brachypodium *stems. Acid pretreatment above these concentrations did not result in increased sugars production after hydrolysis. Conversely, acid pretreatment of paper (used here as a de-lignified control) only marginally increased the release of sugars after hydrolysis, and concentrations of sulphuric acid above 1% did not increase saccharification in paper (Figure 2). The pretreatment of *Brachypodium *segments with 4% H_2_SO_4 _resulted in a lower production of sugars from stem segments after enzymatic hydrolysis, suggesting that a certain proportion of the polysaccharides accessible to the enzymes are released by the acid during the pretreatment (Figure 2).

**Figure 3:**
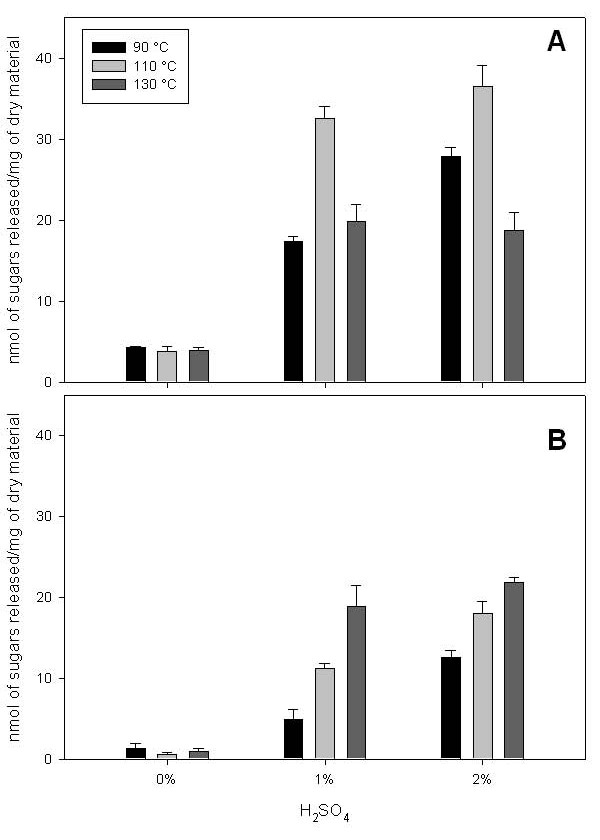
**Sugar production from *Brachypodium *stems pretreated at different temperatures and acid concentrations**. (A) Sugars released after enzymatic treatment of the stem segments. (B) Sugars present in the pretreatment media before enzymatic hydrolysis. Results represent the mean (± SD) of three experiments.

Further effects of different pretreatment conditions were studied, combining increasing temperatures with H_2_SO_4_. The release of sugars by enzymatic hydrolysis remained unaltered in the absence of H_2_SO_4_, irrespective of increase in the temperature (up to 130°C) (Figure 3A). The efficiency of the enzymatic saccharification increased following pretreatment with H_2_SO_4 _at all temperatures, but we found that temperature and H_2_SO_4 _concentration generally interact in a cooperative way leading to increased enzymatic saccharification. However, pretreatment at 130°C in the presence of H_2_SO_4 _led to a lower sugar release on cellulase treatment than that seen at lower temperatures. As shown in Figure 2 for H_2_SO_4 _concentration above 4% at 90°C, this decrease in the efficiency in the release of sugars is probably due to chemical hydrolysis produced by the combination of high acid concentration and high temperature. Indeed, the sugars present in the media of the pretreatment show a drastic increase when the H_2_SO_4 _concentrations and temperature increase (Figure 3B). Hemicellulose hydrolysis has been reported in corn stover under diluted acid conditions and slightly higher temperatures [16]. Under the mild hydrolysis conditions used in the present work, the release of sugars produced during the acid pretreatment limits the availability of substrates for the subsequent enzymatic action. Alternatively, hot acid treatments can lead to the formation of inhibitors of enzyme action and thereby reduce sugar release [17]. In our experiments, this explanation seems unlikely as the segments are rinsed after acid treatment prior to adding the enzymes.

**Figure 4:**
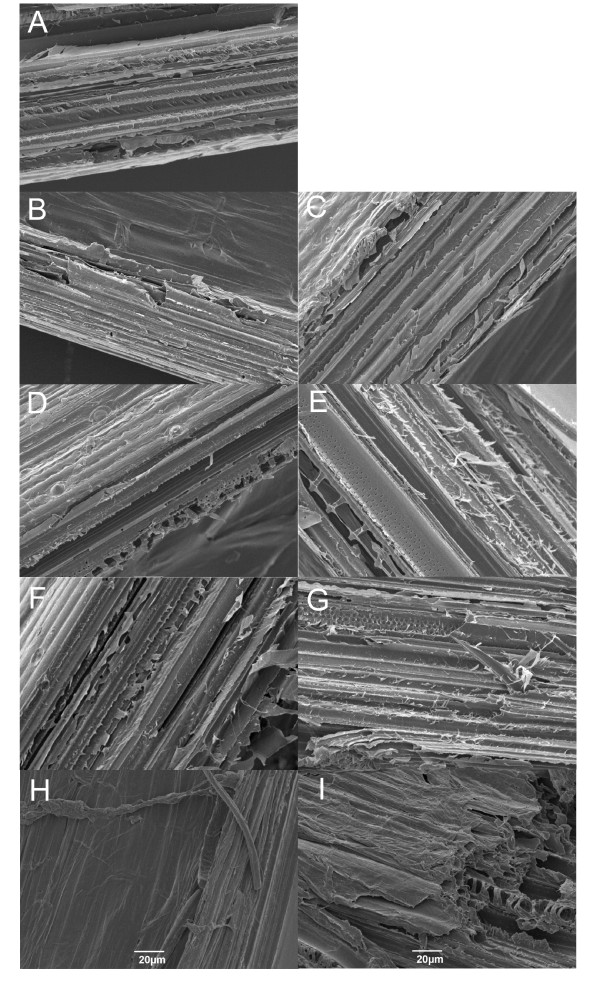
**Scanning electron microscopy showing the effect of different saccharification condition in *Brachypodium *stems**. Images of untreated (A), pretreated only (B, D, F, and H) and pretreated and digested stems (C, E, G, and I). Images B to E show pretreatment at 90°C (B and C without acid and D and E with 2% H_2_SO_4_), and images F to I show pretreatment at 130°C (F and G without acid and H and I with 2% H_2_SO_4_).

The effect of different pretreatment conditions in the structure of the *Brachypodium *stem segments was observed using scanning electron microscopy (SEM). Figure 4 shows sections obtained by splitting the segments longitudinally, in order to observe the vascular elements and epidermis after pretreatment and subsequent enzymatic hydrolysis. Pretreatment at 90°C with or without enzymatic hydrolysis shows no discernable change in the structure of the vascular tissue. After enzymatic treatment the tissue appears slightly disorganised, but no major effect is observed (Figure 4). The pretreatment with 2% H_2_SO_4 _at 90°C also shows no obvious changes in the structure of the tissue, but following subsequent enzymatic hydrolysis there are clear changes in structure with thinner and 'flaky' walls apparent in the longitudinal sections. The longitudinal sections show clear alterations when pretreated at 130°C, even in the absence of H_2_SO_4_. When the segments are pretreated at 130°C in the presence of acid, fibrous material is exposed in the surface of the material (Figure 4). Similar exposure of fibrous cell wall material has been shown in barley hull, particularly at the level of epidermis [18].

### Analysis of sugars released during pretreatment at different temperatures

**Figure 5:**
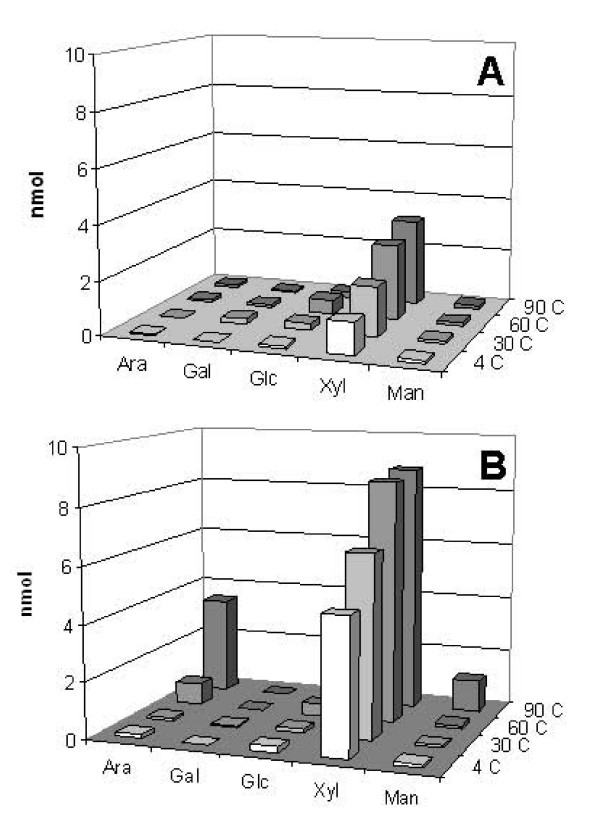
**Analysis of monosaccharides present in the pretreatment media**. *Brachypodium *stem segments were incubated at 4, 30, 60, and 90°C without (A) and with 1% H_2_SO_4 _(B) and the sugars released during pretreatment analysed using a Dionex system (see Methods for details). Results presented here are representative of three repetitions.

In order to study the effect of mild pretreatment with dilute acid, we studied the composition of sugars released after 20 minutes of pretreatment with and without acid and at different temperatures. Figure 5 shows that xylose is the major monosaccharide released during pretreatment without H_2_SO_4 _acid. Small amounts of glucose were also released. Increasing temperature led to increased amounts of xylose and glucose being released, but no other detectable sugars appeared (Figure 5A). When the stem segments were pretreated in the presence of 1% H_2_SO_4 _at 4°C, the predominant sugar detected was xylose with traces of glucose and mannose (Figure 5B). Increasing temperature in the presence of H_2_SO_4 _led to an increase in the release of xylose, mannose and glucose, with arabinose also present in the pretreatment media. Many studies show hemicellulose hydrolysis produced by diluted acid treatment [19,20]. These results suggest that acid pretreatment at high temperatures induces the breakdown of hemicellulose in *Brachypodium *segments, mainly glucuronooarabinoxylans.

### Standard conditions of hydrolysis and effect on *Brachypodium *stems

**Figure 6:**
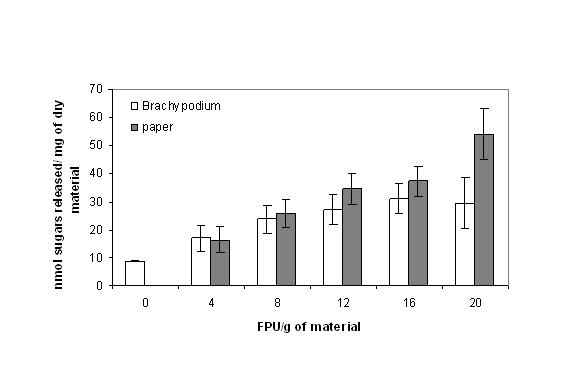
**Effect of increasing amount of enzymes in the digestion of *Brachypodium *stem segments and paper discs**. Paper discs and plant material were pretreated at 90°C with 1% H_2_SO_4 _for 20 min and subsequently hydrolysed with increasing amounts of enzyme. Sugars were determined in the incubation media. Results present the mean (± SD) of triplicate experiments.

The saccharification of *Brachypodium *stems was compared with the digestion of paper in order to compare the dynamics of sugar release from cellulosic and lignocellulosic materials. An increasing amount of enzyme progressively increased the release of sugars in paper (Figure 6), showing that no structural barriers limit the enzymatic activity in this case. In contrast, in *Brachypodium *stems, the release of sugars increased with increasing enzyme load, to 12 filter paper units (FPU)/g and then stabilised (Figure 6), suggesting that access to substrates is more limited than is the case with paper.

**Figure 7:**
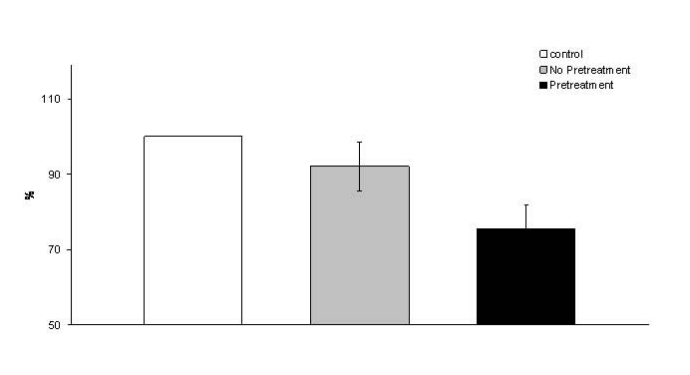
**Total sugars remaining in stem segments after pretreatment and hydrolysis**. Segments were subjected to complete hydrolysis after pretreatment only (1% H_2_SO_4_, 20 min at 90°C) or pretreatment and enzymatic hydrolysis (4 FPU/g of material). Total sugars were determined using MBTH using glucose as standard. Results represent the mean (± SD) of three experiments.

One of the major aims of the work presented here is to establish conditions for use in high-throughput screens to enable the identification of alterations in saccharification potential in mutant populations. In contrast to industrial processing where maximum release of sugars is desirable, we wish to identify mild pretreatment conditions that allow the enzymes to release limited quantities of sugar from wild type stems and will enable changes in the ease of sugar mobilisation with enzymes in potential mutants to be detected. In a typical digestion we pretreat 4 mm whole stem segments with 1% H_2_SO_4 _at 90°C for 20 min. Subsequent digestion of the pretreated material is carried out for 1 h at 30°C, using an enzyme cocktail containing 4 FPU and 19 cellobiose units (CBU) (see Methods for details) per gram of material. Under these conditions, an average of 75% of the total sugars in the material remained undigested in the stems following pretreatment and enzyme hydrolysis, whilst 90% remained following enzyme hydrolysis without pretreatment (Figure 7). SEM imaging showed minor structural alterations of the *Brachypodium *stems following hydrolysis (Figure 4).

### Time course of hydrolysis and products released during enzymatic digestion

**Figure 8:**
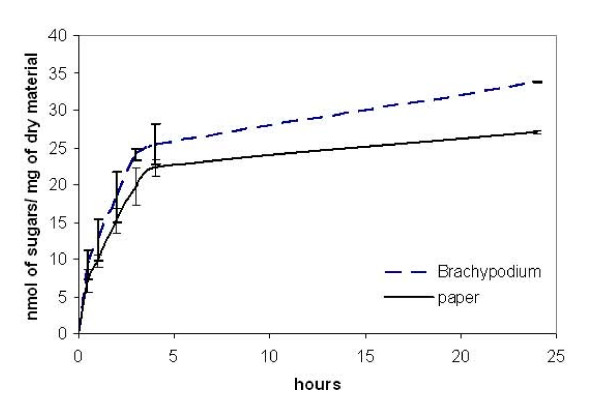
**Time course of hydrolysis**. Paper discs and stem segments were pretreated (1% H_2_SO_4_, 20 min at 90°C) and then incubated with a cellulase mixture (4 FPU/g of material) over a period of 24 h. Sugars were quantified as described in Methods. Results represent the mean (± SD) of three experiments.

Using an enzyme concentration 4 FPU/g of *Brachypodium *stems, the enzymatic hydrolysis proceeded at maximum rate over the first 4 h, after which the rate declined (Figure 8). A similar trend was seen with paper used as a substrate.

**Figure 9:**
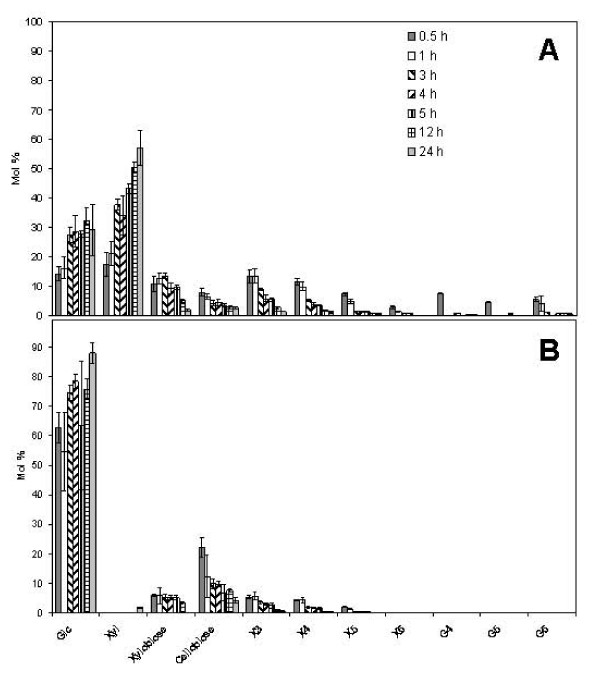
**Analysis of the oligosaccharides released by enzymatic digestion of *Brachypodium *stem segments and papers discs over a 24 h period of incubation**. Materials were digested as described for the time course of the incubation (Figure 8 and Methods) At the indicated times, samples were analysed using high performance anion-exchange chromatography. Standards for the glucose and xylose oligosaccharide series were for quantification. Panel A represents the oligosaccharides present in the incubation media of *Brachypodium *stems and panel B the results for paper discs. Glc: glucose; G4: cellotetraose; G5: cellopentaose; G6 cellohexaose; Xyl: xylose; X3:xylotriose; X4: xylotetraose; X5: xylopentaose. Results represent the mean (± SD) of three experiments.

Analyses of the products released during 24 h digestion revealed a gradual accumulation of monosaccharides, whilst oligosaccharide species were relatively abundant early in the digestion but disappear with time (Figure 9). The digestion products from paper were dominated by glucose and cello-oligomers, whereas in the digestion products from *Brachypodium*, xylose and xylo-oligosaccharides predominated over gluco- and cello-oligosaccharides. This most likely reflects the fact that paper has already seen substantial loss of hemicelluloses during the pulping process, and that pulping has also produced cleaner cellulose fibres more readily attacked by cellulases. Although xylose was not detected in the oligosaccharide analysis (Figure 9A), monosaccharide analyses of the digestion products from paper show that xylose is present after enzyme action, but the low amounts released (relative to glucose) do not allow quantification under the analytical conditions of the oligosaccharide analyses (data not shown).

In contrast, the *Brachypodium *stems have only been exposed to the gentle pretreatment conditions used in our assay and hence the more readily digestible hemicellulose components are releasing greater quantities of sugar than the more recalcitrant cellulose (Figure 9B). Saccharification analysis of dilute H_2_SO_4_-impregnated wheat straw produced large amounts of xylose after pretreatment in enzymatic hydrolysis [21]. Since some of the main candidate energy crops are grasses, high levels of xylose are expected to be released for fermentation, underlying the need for pentose-fermenting organisms in the production of liquid biofuels [22].

## Conclusion

*Brachypodium *is being considered as a suitable experimental model species to investigate various parameters related to the use of fast-growing biomass grasses in the context of biofuels and bioenergy [3,6]. Work presented in this paper shows that the composition of non-cellulosic polysaccharides in *Brachypodium *appears broadly similar to that of biomass grasses and cereals, and that all of these differ to that seen in *Arabidopsis*, indicating that from a perspective of cell wall composition *Brachypodium *appears to be a suitable model. In order to underpin the development of high-throughput saccharification screens that can be used to identify *Brachypodium *mutants with altered digestibility characteristics, we have undertaken an examination into the effects of a range of pretreatment and enzymatic hydrolysis conditions. Dilute acid pretreatment has been shown to be an efficient method of increasing efficiency in the digestion of lignocellulosic materials in general. Here we show the effect of particular combinations of pretreatment and enzymatic hydrolysis in the saccharification of *Brachypodium *stems.

We found that both pretreatment temperature and acid concentration had effects on subsequent enzymatic saccharification. Temperatures above 110°C combined with acid in the pretreatment led to hydrolysis of cell wall polysaccharides directly, decreasing the amount of sugars released by subsequent enzymatic hydrolysis. The monosaccharide composition of the sugars released during pretreatment corresponds to hemicellulosic materials. SEM images of the segments after pretreatment and enzymatic attack revealed changes in stem structure associated with changes in sugar release. Our data support the use of *Brachypodium *as a model species for studying lignocellulose from cereals and biomass grasses, and suggest a combination of modest temperature and dilute acid pretreatment may form the basis of a sensitive screening method to identify genes affecting grass biomass saccharification potential.

## Methods

### Plant materials and growth conditions

Seed of *Brachypodium distachyon *Bd21 were sown in 4-inch pots, watered, and kept at 4°C for two weeks prior to growth. Plants were grown in a glasshouse under a 16-hours light regime, at 22°C. After setting seed, plants were left to dry in the pots. For studying saccharification, stem segments of 4 mm length were taken from the middle section of internodes 5, 6, and 7. Preliminary studies showed that there were no differences in saccharification using material taken from these internodes. In all the saccharification assays 4 mg samples were used and assays were carried out in quadruplicates. In the case of paper used for comparative purposes with *Brachypodium *stems, samples were 4 mg of 5 mm discs of 80 g/m^2 ^recycled paper (M-Real, Amsterdam, The Netherlands).

### Hydrolysis of materials

Enzymatic hydrolysis was carried out using Celluclast (cellulase from *Trichoderma reesei*) and Novozyme 188 (cellobiase from *Aspergillus niger*) in an enzyme cocktail containing a 4:1 proportion of Celluclast: Novozyme 188. The enzymes were filtered using a Hi-Trap desalting column (GE Healthcare, Little Chalfont, UK) before use and the hydrolysis was carried out during the indicated times, at 30°C in buffer Na Acetate 25 mM at pH 4.5.

### Total sugar content

The determination of total sugar content in untreated, pretreated, and no pretreatment stems was carried out following the protocol described by Fry [23]. 4 mg of stem fragments were dissolved in 72% H_2_SO_4 _at 30°C and then hydrolysed in 4% H_2_SO_4 _at 120°C for 1 h. The resulting solution was neutralised with Ba(OH)_2 _and the sugars were measured as described below in sugar determination.

### Scanning electro microscopy

Stems were incubated in buffer Na Acetate 25 mM pH 4.5 for 20 min at room temperature (untreated and no pretreatment), or incubated in 1% H_2_SO_4 _for 20 min at 90°C (pretreated). The segments were rinsed several times with buffer and then incubated with 0 or 0.5 mg of enzyme cocktail for 1 h. Segments were placed in 100% ethanol and then air-dried. For SEM, stems were mounted in SEM stubs and coated with gold/palladium. The mounted specimens were observed with a JEOL JSM 6490LV (Jeol Ltd, Tokyo, Japan) at an accelerating voltage of 5kV.

### Sugar determination

The determination of sugars released after hydrolysis was done using a modification of the method by Anton and Barrett [24] using 3-methyl-2-benzothiazolinone hydrozone (MTBH). Determination was carried out in a 1 ml final volume using 300 μl of sample. The detection reaction contained 0.25 N NaOH, 0.06% MTBH, 0.02% DTT, and colour was developed by adding 0.2% FeNH_4_(SO_4_)_2_, 0.2% Sulfamic acid and 0.1% HCl. The absorbance of the reaction was followed at 620 nm. This method was tested for the detection of a range of sugars that are released from the cell wall, showing no significant differences between reducing sugars.

### Sugar analysis

Oligosaccharide analyses of the sugars released from stem segments after hydrolysis was carried out by taking 0.2 ml samples from digestions after 0.5, 1, 3, 4, 5, 12, and 24 h of enzymatic digestion. Samples were filtered using AG50W × 12 resin (Bio-Rad Laboratories Ltd, Hemel Hempstead, UK) and subsequently dried and re-suspended in 100 μl of deionised water. The samples were filtered using a Millex LH 0.45 μm pored filter (Millipore, Billerica, USA) and then separated by high-performance anion-exchange chromatography (HPAEC) on a Dionex Carbopac PA-100 column (Dionex Corporation, Sunnyvale, USA) with integrated amperometry detection. The separated oligosaccharides were quantified by using external calibration with an equimolar mixture of eleven monosaccharide and oligosaccharide standards (glucose, cellobiose, cellotriose, cellotetraose, cellopentaose, cellohexaose, xylose, xylobiose, xylotriose, xylotetraose, and xylopentaose).

Cell walls for non-cellulosic sugar composition from grasses and *Arabidopsis *were extracted from mature stems. Tissue was subsequently extracted with phenol, chlorophorm:methanol 4:1, 90% DMSO and 100% ethanol. 4 mg of dried material was digested with 2 M trifluoroacetic acid at 100°C for 4 h. After the acid was evaporated, samples were rinsed with isopropanol and resuspended in 100 μl of deionised water. Samples were filtered and analysed as described below.

For the determination of sugars during pretreatment, samples were taken after 20 min of pretreatment in the presence of 0 and 1% H_2_SO_4_at 4, 30, 60 and 90°C. Monosaccharide analyses were performed by HPAEC using a Dionex Carbopac PA-10 as described in [25]. Samples (1.4 ml) were collected immediately after pretreatment and filtered using a column of Dowex 1 × 2 to eliminate the H_2_SO_4 _from the samples. The monosaccharide standards used for quantification were arabinose, fucose, galactose, galacturonic acid, glucose, glucuronic acid, mannose, rhamnose, and xylose.

## Competing interests

The authors declare that they have no competing interests.

## Authors' contributions

LDG carried out the saccharification assays, monosaccharide analysis, preparation of materials, supervised the work in the laboratory and contributed to the manuscript preparation. JKB carried out the oligosaccharide analysis and participated in determining the saccharification conditions. ERS undertook the SEM work and characterisation of pretreatments. SJMM helped in the experimental design, participated in the saccharification analysis, contributed to the manuscript preparation, and obtained funding for the work. All authors read and approved the final manuscript.
